# Biochemical Evaluation of Green-Synthesized Silver Nanoparticles Derived from Waste *Eriobotrya Japonica* Seeds: Enzyme Inhibition and Antiglycation Potential

**DOI:** 10.1007/s12010-026-05775-x

**Published:** 2026-06-09

**Authors:** Serap Savci, Ebru Kocadag Kocazorbaz

**Affiliations:** Faculty of Science, Department of Biochemistry, Ege University, Izmir, Turkey

**Keywords:** Silver nanoparticles, Alpha-glucosidase inhibition, Dipeptidyl peptidase-IV inhibition, Antiglycation activity, Bioactive nanomaterials

## Abstract

Silver nanoparticles were synthesized via a phytochemical-mediated green approach using waste *Eriobotrya japonica* seed extract as a natural reducing and stabilizing agent. This eco-friendly strategy eliminated the need for hazardous chemical reductants by utilizing bioactive phytochemicals present in the seed matrix. Nanoparticle formation was confirmed by ultraviolet–visible spectroscopy through a characteristic surface plasmon resonance band. Physicochemical characterization was performed using Fourier transform infrared spectroscopy, X-ray diffraction, dynamic light scattering, zeta potential analysis, scanning electron microscopy, and energy-dispersive X-ray spectroscopy. The X-ray diffraction pattern displayed distinct reflections at 2θ values of 38.2°, 44.4°, 64.6°, and 77.5°, corresponding to the face-centered cubic crystalline structure of metallic silver, while peak broadening indicated nanoscale crystallite formation. Scanning electron microscopy revealed predominantly spherical nanoparticles with an average size of 13.52 nm. The zeta potential value of − 23.7 ± 7.50 mV suggested moderate colloidal stability and effective phytochemical surface stabilization. The biological activities of the synthesized nanoparticles were evaluated in comparison with the crude extract. The nanoparticles exhibited enhanced inhibitory effects against α-glucosidase and dipeptidyl peptidase-IV enzymes, along with notable antiglycation activity. The half-maximal inhibitory concentration for antiglycation was 11.04 µg/mL, substantially lower than that of the seed extract (164.9 µg/mL), indicating improved biofunctional performance following nanoparticle synthesis. These findings suggest that waste loquat seeds can be effectively converted into bioactive silver nanoparticles with potential relevance in antioxidant, antiglycation, and enzyme inhibitory applications. The present results are limited to in vitro evaluations, and the safety profile and underlying mechanisms remain to be clarified.

## Introduction

Nanotechnology has attracted increasing interest due to the unique properties and wide range of applications of nanomaterials, and various chemical and physical methods have been employed for their synthesis [1]. However, these conventional methods often involve high energy consumption and the use of hazardous chemicals. In contrast, green synthesis offers an environmentally friendly and cost-effective alternative by utilizing biological systems for the reduction of metal precursors into nanoparticles [2, 3]. In recent years, the biological synthesis of nanoparticles using algae, yeast, fungi, bacteria, other microorganisms, and different plant parts such as roots, stems, seeds, and leaves has gained significant attention [4]. Green synthesis relies on simple, non-toxic, and renewable natural materials, making it a sustainable approach within the framework of green chemistry. This method is preferred due to its advantages, including energy efficiency, waste minimization, ease of application, and the elimination of harmful chemical reagents [5]. Nanoparticles produced via biological routes are particularly suitable for biomedical applications, as they exhibit enhanced biocompatibility and reduced toxicity [6]. In particular, noble metal nanoparticles such as silver and gold have attracted considerable attention due to their unique physicochemical properties and wide range of biomedical applications. These nanoparticles have been extensively explored for their antimicrobial, antioxidant, anticancer, and enzyme inhibitory activities, as well as their potential use in drug delivery and diagnostic systems. Recent review studies have highlighted the growing importance of silver and gold nanoparticles in nanomedicine and their potential in developing advanced therapeutic strategies [7, 8].Plant seeds are especially rich in secondary metabolites that can act as effective reducing and stabilizing agents, making them promising raw materials for the green synthesis of nanoparticles [9].

In general, the utilization of waste represents a fundamental sustainable and resource-efficient approach. In recent years, waste generation has increased significantly due to population growth and intensified industrial activities [10]. The uncontrolled accumulation or improper disposal of waste causes serious problems, including environmental pollution, depletion of valuable resources, and adverse effects on human health [11, 12]Therefore, the effective management and valorization of waste are critical for achieving a sustainable future [13, 14] In addition to generating economic value, proper waste utilization reduces the consumption of natural resources, conserves energy, and minimizes environmental pollution. Every year, large quantities of fruit peels and seeds are generated worldwide and are commonly treated as waste, leading to both environmental burdens and the loss of potentially valuable resources [15, 16] Notably, waste fruit seeds are rich in phytochemicals, which makes them a.

promising renewable resource. Phytochemicals have been reported to play an important role in the prevention and management of various health problems, including cancer, cardiovascular diseases, hypertension, hormonal disorders, and diabetes [17, 18].

Diabetes is a multifactorial metabolic disorder, and its rapidly increasing prevalence worldwide has intensified the search for new preventive and therapeutic strategies [19]. Prolonged exposure to hyperglycemia leads to non-enzymatic glycation, glyco-oxidation, and protein aggregation, which play a crucial role in the development of diabetes-related complications. These processes contribute to the pathogenesis of aging and several aging-associated diseases, including Alzheimer’s disease, rheumatoid arthritis, neurodegenerative disorders, cancer, and cataracts [20]. Consequently, the normalization of hyperglycemia through enzyme inhibition, as well as the prevention of non-enzymatic glycation and the formation of advanced glycation end products (AGEs), has attracted considerable attention in recent years. There is growing interest in natural compounds derived from various biological sources that may exert antidiabetic effects through multiple mechanisms. Due to the side effects associated with many synthetic drugs, natural therapeutic agents with fewer or no adverse effects have gained increasing attention. In particular, edible and medicinal plants, which have played a vital role in human nutrition and traditional medicine since ancient times, are being extensively investigated [21, 22].

*Eriobotrya japonica* L. (loquat) is a tree species belonging to the Rosaceae family [23]. Loquat fruits are widely used for the production of products such as wine, syrup, vinegar, and marmalade, generating large amounts of waste in the form of peels and seeds. Loquat fruits typically contain five large brown seeds, which constitute more than half of the total fruit volume As loquat production increases, the amount of seed waste generated also rises [24, 25]. Despite being commonly discarded, loquat seeds represent a promising raw material for green synthesis due to their rich phytochemical profile. These seeds contain significant amounts of phenolic compounds, flavonoids, and other bioactive constituents that can function as natural reducing and stabilizing agents in nanoparticle synthesis. Therefore, the utilization of loquat seed extract provides a dual advantage by enabling the eco-friendly synthesis of nanoparticles while simultaneously contributing to the valorization of agricultural waste. Loquat seeds are rich in starch, unsaturated fatty acids (linolenic and linoleic acids), β-sitosterol, and polyphenolic compounds. High concentrations of bioactive compounds, including phenolic acids and flavonoids, have been reported to exhibit potential therapeutic effects, particularly in neurodegenerative diseases, by reducing neuroinflammation, oxidative stress, and β-amyloid aggregation [26]. In this study, for the first time, waste loquat seeds were utilized as a plant-based resource for the green synthesis of silver nanoparticles. The synthesized nanoparticles were characterized using dynamic light scattering (DLS), zeta potential analysis, Fourier-transform infrared spectroscopy (FTIR), X-ray diffraction (XRD), scanning electron microscopy (SEM), and UV–visible spectroscopy. Furthermore, the antioxidant, antiglycation, and antidiabetic activities of the synthesized nanoparticles were systematically evaluated.

## Materials and Methods

### Preparation and Extraction of *Eriobotrya Japonica* Seed Flour

Loquat (Eriobotrya japonica L.) fruits used in this study were collected from the Izmir region (Türkiye) in June. The seeds were manually separated from the fruits and dried completely under shaded conditions at ambient temperature, avoiding direct sunlight. The dried seeds were ground into a fine powder using a laboratory-scale blender. To remove lipophilic components, the powdered loquat seed material was defatted by mixing with n-hexane at a ratio of 1:5 (w/v) under continuous stirring for 3 h at room temperature. Following defatting, the mixture was subjected to vacuum filtration to remove the solvent. The defatted seed powder was then air-dried overnight to ensure complete evaporation of residual n-hexane. For aqueous extraction, the dried seed flour was extracted with distilled water in a shaking water bath for 12 h at room temperature. The resulting mixture was centrifuged at 9000 rpm for 30 min, and the supernatant was carefully collected and filtered through Whatman No. 1 filter paper. The obtained loquat seed flour extract was stored at 4 °C until further use.

### Determination of Total Phenolic Content

The total phenolic content (TPC) of the loquat seed extracts was determined using the Folin–Ciocalteu method, as described by Singleton et al. [27]. Briefly, 200 µL of the seed extract was mixed with 1000 µL of diluted Folin–Ciocalteu reagent and incubated for 5 min at room temperature. Subsequently, 800 µL of sodium carbonate solution (7.5%, w/v) was added to the mixture with vigorous stirring, and the reaction was allowed to proceed for 1 h in the dark. The absorbance of the resulting solutions was measured at 760 nm using a UV–visible spectrophotometer. Gallic acid solutions at different concentrations were used to construct a calibration curve. The total phenolic content of the samples was expressed as micrograms of gallic acid equivalents per gram of sample (µg GAE/g). All measurements were performed in triplicate.

### Determination of Total Flavonoid Content

The total flavonoid content (TFC) of the loquat seed extract was determined according to the method described by Meda et al. [28]. Briefly, 100 µL of the extract was mixed with 100 µL of a 2% (w/v) aluminum chloride (AlCl_3_·6H_2_O) solution prepared in methanol. The reaction mixture was incubated for 10 min at room temperature. The absorbance was measured at 405 nm using a microplate reader (Thermo Scientific, Multiskan FC Microplate Photometer). Quercetin solutions at different concentrations were used to generate the calibration curve. The total flavonoid content was expressed as milligrams of quercetin equivalents per gram of extract (mg QE/g). All analyses were performed in triplicate.

### Green Synthesis of Silver Nanoparticles from *Eriobotrya Japonica* Waste Seeds

Silver nanoparticles (AgNPs) were synthesized using an green synthesis approach with *Eriobotrya japonica* waste seed extract as the reducing and stabilizing agent. Briefly, 100 mL of the aqueous *E. japonica s*eed extract was added dropwise to 100 mL of 6 mM silver nitrate (AgNO_3_) solution under continuous magnetic stirring at 150 rpm. The reaction mixture was maintained at 50 °C for 4 h. The formation of AgNPs was initially confirmed by a visible color change of the reaction mixture from light yellow to bronze brown, indicating the reduction of Ag^+^ ions to metallic silver. After completion of the reaction, the mixture was centrifuged at 15.000 rpm for 30 min. The resulting pellet containing AgNPs was washed three times with deionized water and once with ethanol to remove unreacted compounds and residual biomolecules. Finally, the purified nanoparticles were dried in a hot air oven at 70 °C and stored for further characterization and biological assays.

### Characterization of Silver Nanoparticles

The optical properties of the synthesized silver nanoparticles (AgNPs) were analyzed using UV–visible spectroscopy (Thermo Multiscan 1500) by recording the absorption spectra in the wavelength range of 200–800 nm. The hydrodynamic size distribution and surface charge (zeta potential) of the AgNPs were determined using a Zetasizer instrument (Malvern ZS). Fourier-transform infrared spectroscopy (FTIR; PerkinElmer, Spectrum Two) was employed to identify the functional groups involved in the reduction and stabilization of the nanoparticles. The crystalline structure of the AgNPs was examined by X-ray diffraction (XRD) analysis using a Rigaku MiniFlex diffractometer. The morphology, particle size, and surface characteristics of the AgNPs were observed using scanning electron microscopy (SEM; LEO 1430 VP).

### Antioxidant Activity (DPPH Radical Scavenging Assay)

The antioxidant activity of the loquat seed extract and the synthesized silver nanoparticle solution was evaluated using the 2,2-diphenyl-1-picrylhydrazyl (DPPH) radical scavenging assay. Briefly, equal volumes (1.0 mL) of the sample and DPPH solution prepared in 80% methanol were mixed and incubated at 37 °C for 30 min in the dark. Following incubation, the absorbance of the reaction mixture was measured at 517 nm using a UV–visible spectrophotometer. A calibration curve was constructed using Trolox as the standard antioxidant. The antioxidant capacity of the samples was expressed as milligrams of Trolox equivalent antioxidant capacity per gram of dry matter (mg TEAC/g DM). All measurements were performed in triplicate.

### α-Glucosidase Inhibitory Activity

The α-glucosidase inhibitory activity of the synthesized *Eriobotrya japonica*–derived silver nanoparticles (EJ-NPs) was evaluated using a microplate-based assay [29]. Briefly, 36 µL of phosphate buffer solution was mixed with 30 µL of EJ-NPs solution at various concentrations (200–1000 µg/mL) in each well. Subsequently, 17 µL of α-glucosidase enzyme solution (0.15 U/mL) was added, and the mixture was pre-incubated at 37 °C for 5 min. After pre-incubation, 17 µL of 4-nitrophenyl-α-D-glucopyranoside (pNPG) substrate solution (2 mM) was added to initiate the enzymatic reaction, resulting in a final reaction volume of 100 µL per well. The reaction was allowed to proceed for 15 min. at 37 °C and was terminated by the addition of 100 µL of sodium carbonate solution (200 mM). The absorbance was measured at 405 nm using a microplate reader. All assays were performed in triplicate.

### Dipeptidyl Peptidase-IV (DPP-IV) Inhibitory Activity

The DPP-IV inhibitory activity of the synthesized EJ-NPs was determined according to a previously reported microplate assay with minor modifications [30]. Briefly, 75 µL of Tris–HCl buffer (pH 8.0) was mixed with 25 µL of EJ-NPs solution at different concentrations in each well. Subsequently, 50 µL of DPP-IV enzyme solution was added, and the mixture was pre-incubated at 37 °C for 10 min. The enzymatic reaction was initiated by the addition of 100 µL of 2mM Gly-Pro-p-nitroanilide (Gly-Pro-pNA) substrate solution. The reaction mixture was incubated at 37 °C for 30 min, and the release of p-nitroaniline was monitored by measuring the absorbance at 405 nm using a microplate reader. The percentage inhibition of DPP-IV activity was calculated relative to the control without inhibitor. All experiments were performed in triplicate.

### Antiglycation Activity

The antiglycation activity of the EJ-NPs was evaluated using a bovine serum albumin (BSA)–sugar model, following a previously reported method [31]. Briefly, the reaction mixture consisted of BSA solution (10 mg/mL), glucose (0.5 M), and fructose (0.5 M) prepared in phosphate buffer (0.1 M, pH 7.4). EJ-NPs at different concentrations were added to the reaction mixture, and the final volume was adjusted with phosphate buffer. The samples were incubated at 60 °C for 2 h under sterile conditions. After incubation, the formation of advanced glycation end products (AGEs) was evaluated by measuring fluorescence intensity at an excitation wavelength of 370 nm and an emission wavelength of 440 nm using a microplate reader. Aminoguanidine was used as a positive control inhibitor. The percentage inhibition of AGE formation was calculated relative to the control without inhibitor. All experiments were performed in triplicate.

## Results and Discussion

### Total Phenolic Content and Flavanoid of *E.japonica* Seed

The total phenolic content of the loquat seed extract was determined using the Folin–Ciocalteu method. The total phenolic content of the loquat seed extract was found to be 16.30 ± 0.57 mg GAE/g seed. The total flavonoid content of the waste loquat seed extract was determined as 2.05 ± 0.01 mg CE/g seed. Due to the presence of aromatic rings, phenolic compounds and flavonoids exhibit strong nucleophilic characteristics and possess significant metal-chelating potential [32]. The phenolic content detected in the green-synthesized AgNPs suggests that the nanoparticles are effectively capped with phenolic compounds derived from the plant extract, which act as both reducing and stabilizing agents during nanoparticle formation.

### Green Synthesis and UV–visible Spectroscopic Analysis of Silver Nanoparticles

**Fig. 1:**
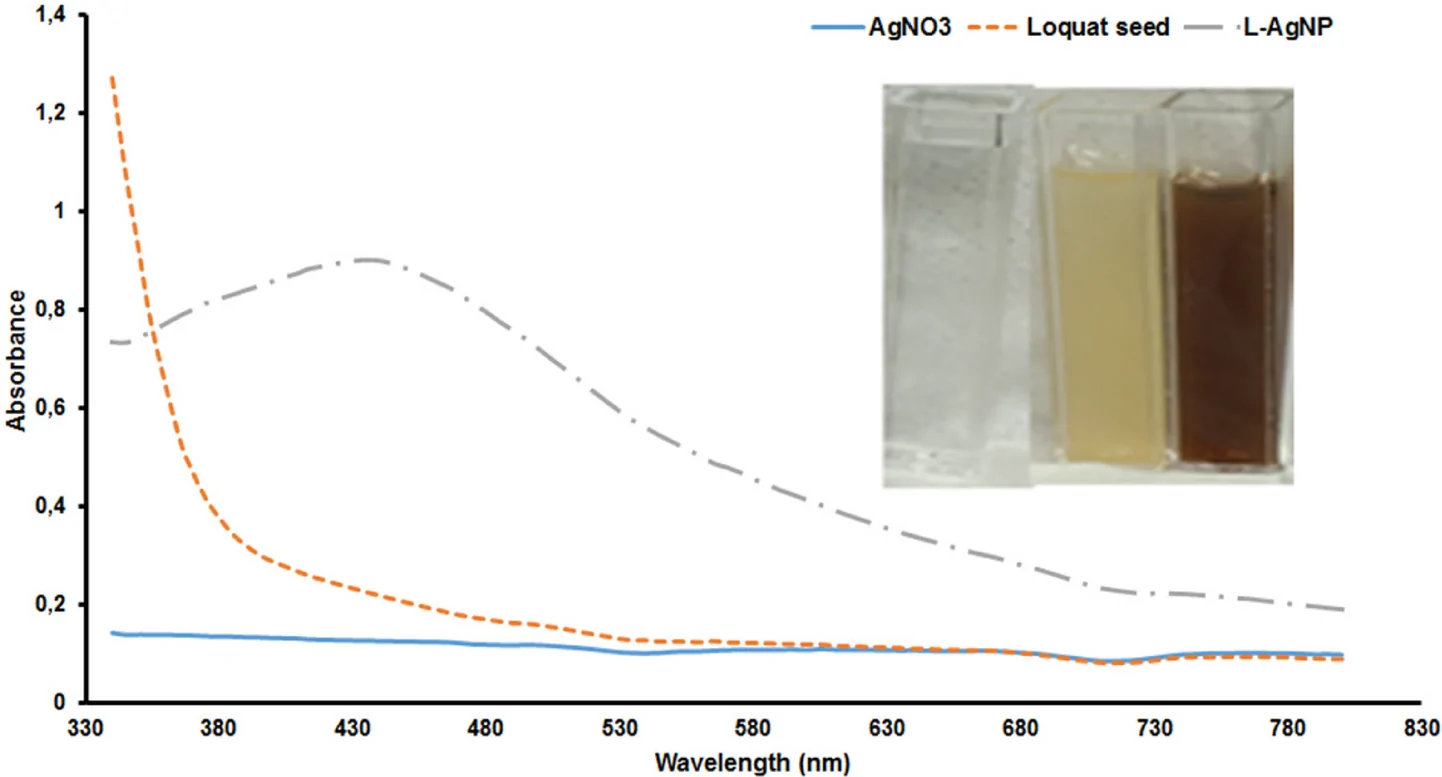
UV–visible spectrum of silver nanoparticles from waste seed aqueous extract of *E. japonica*

The addition of *Eriobotrya japonica* seed extract to a 6 mM AgNO3 solution initiated the formation of silver nanoparticles. As shown in Fig. 1, the color of the AgNO3/*E. japonica* reaction mixture gradually changed from colorless to yellow and subsequently to reddish-brown, indicating the successful synthesis of silver nanoparticles. The formation of nanoparticles in the aqueous solution was further confirmed using UV–visible spectroscopy. The absorbance spectra were recorded in the wavelength range of 340–600 nm. A distinct maximum absorbance peak was observed at 440 nm for the *E. japonica* seed-mediated AgNPs (EJ-AgNPs), which corresponds to the surface plasmon resonance (SPR) of silver nanoparticles. The position and intensity of the SPR band are influenced by the size, shape, and interparticle distance of the nanoparticles [33]. A comparable SPR band has also been reported in a previous study by Rani et al. [34]. The UV–visible spectra of *E. japonica* seed extract and the synthesized AgNPs are presented in Fig. 1.

### FTIR Analysis of Biosynthesized EJ-AgNPs

**Fig. 2:**
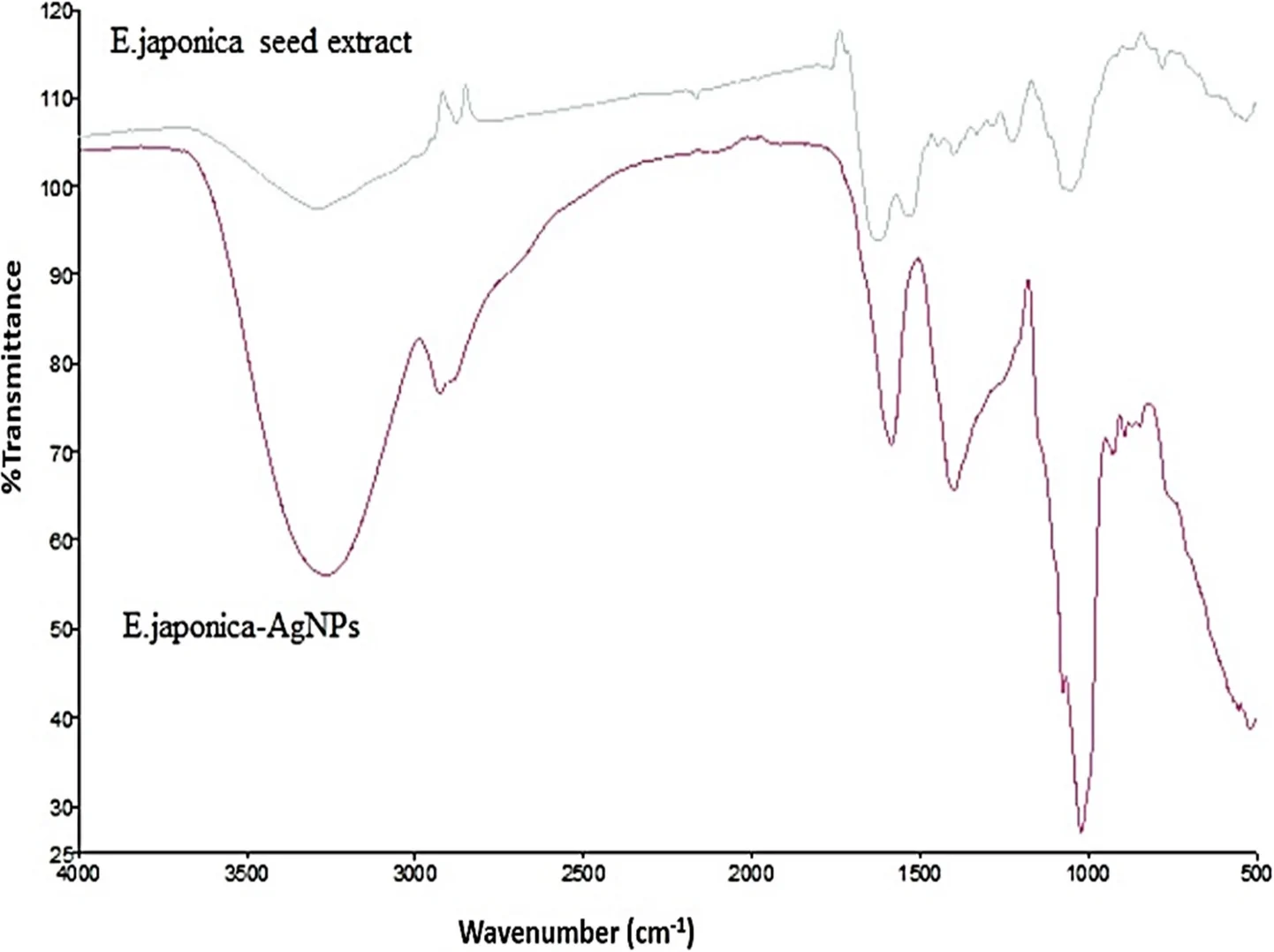
FTIR analysis of green-synthesized EJ-AgNPs

FTIR analysis determined the presence of various functional groups in the molecules responsible for the biological reduction and stabilisation of AgNPs, whether the structure is aromatic or aliphatic, the state of the bonds in the structure and the bonding sites. The FTIR spectrum of silver nitrate nanoparticles synthesized from loquat waste seeds and loquat waste seeds extract are shown in Fig. 2. In the spectrum of EJ-AgNPs, a broad and intense band observed around 3279 cm⁻¹ is attributed to O–H stretching vibrations, indicating the presence of hydroxyl groups commonly associated with phenolic compounds. The peak at 2878 cm⁻¹ corresponds to C–H stretching vibrations of aliphatic groups [35]. Additionally, the absorption bands at 1634 cm⁻¹ and 1400 cm⁻¹ can be assigned to the asymmetric and symmetric stretching vibrations of carbonyl (C = O) and/or carboxylate groups. These functional groups are likely derived from bioactive phytochemicals present in the loquat seed extract, such as phenolic acids and flavonoids [36]. The presence of these functional groups suggests that they may play an important role in the reduction of Ag⁺ ions to Ag⁰ nanoparticles and in the subsequent stabilization of the nanoparticles through surface capping.

### Particle Size Distribution and Zeta Potential of EJ-AgNPs

**Fig. 3:**
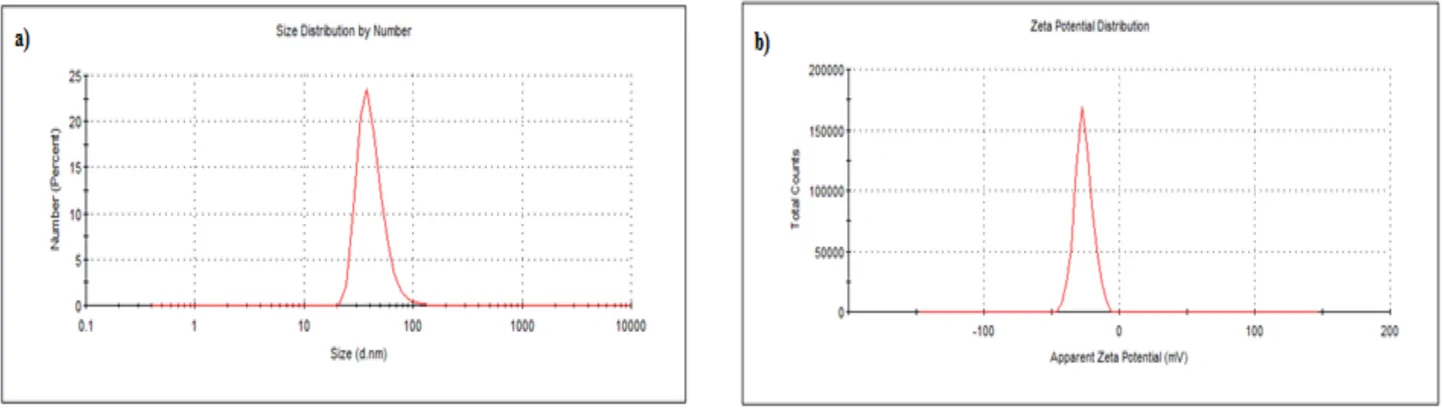
Particle size distribution (**a**) and zeta potential (**b**) of EJ-AgNPs

The particle size distribution of EJ-AgNPs synthesized from waste loquat seed extract, as determined by the dynamic light scattering (DLS) method, is presented in Fig. 3. According to the DLS analysis, the average particle size of the silver nanoparticles synthesized using *E. japonica* seed extract was approximately 83 nm. In addition, the polydispersity index (PDI) value was determined to be 0.578, indicating a moderately broad particle size distribution and suggesting a polydisperse colloidal system. Such polydispersity is commonly observed in plant-mediated green synthesis approaches due to the heterogeneous nature of phytochemical reducing and stabilizing agents present in plant extracts. The zeta potential of the colloidal suspension of green-synthesized EJ-AgNPs is also shown in Fig. 3. The zeta potential value of the nanoparticles was recorded as − 23.7 ± 7.50 mV. Zeta potential is an important parameter indicating the degree of electrostatic repulsion between charged particles and is commonly used to assess the physical stability of nanoparticle suspensions. Strong interparticle repulsive forces prevent nanoparticle agglomeration and contribute to colloidal stability [37]. The negative surface charge of the synthesized EJ-AgNPs is attributed to the reducing and capping agents present in the waste loquat seed extract. The observed negative zeta potential further confirms the presence of significant electrostatic interactions on the nanoparticle surface. The obtained zeta potential value suggests moderate colloidal stability and indicates the presence of electrostatic interactions that may help reduce nanoparticle aggregation [38, 39].

### SEM-EDX Analysis of EJ-AgNPs

**Fig. 4:**
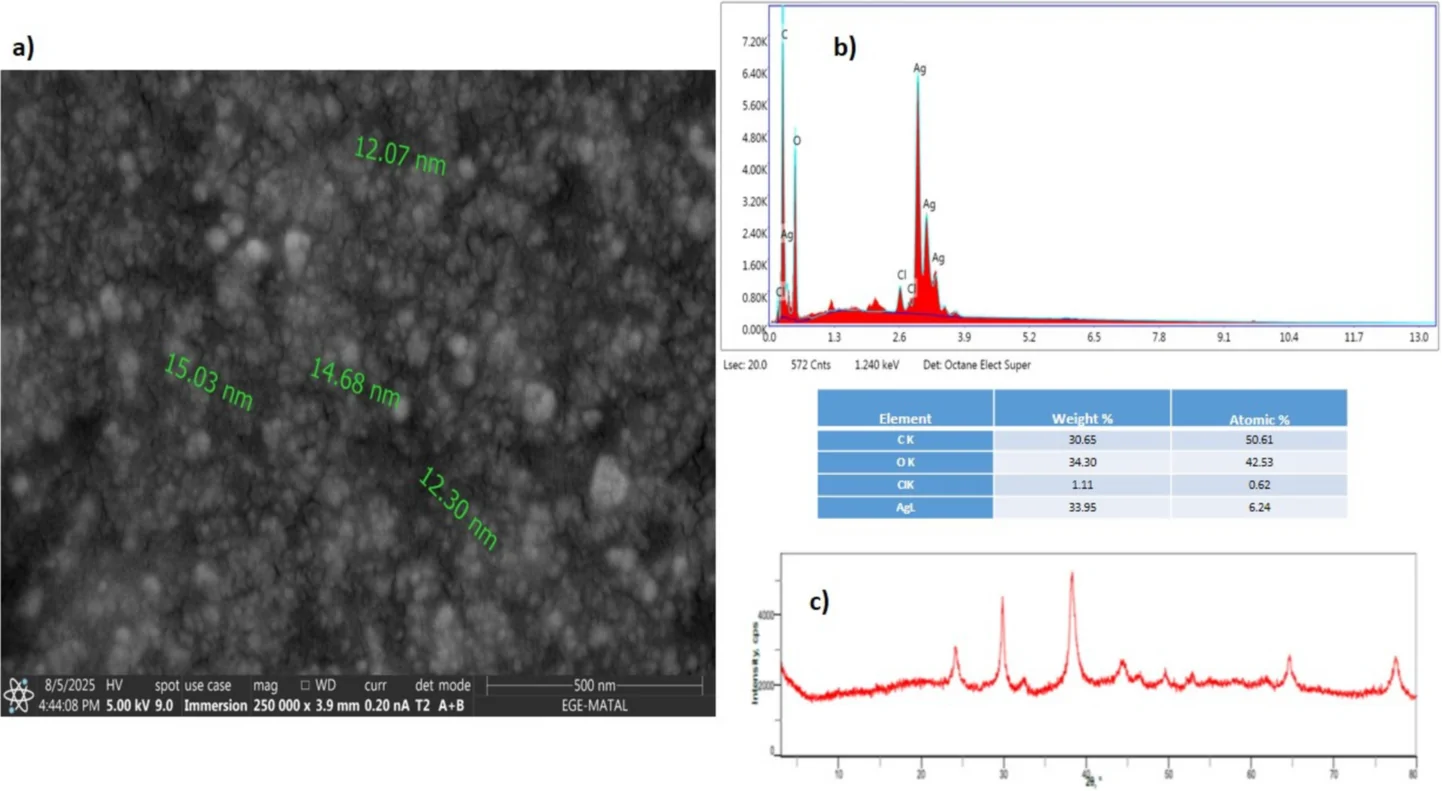
SEM micrographs (**a**) EDX spectrum (**b**) and XRD analysis (**c**) of green-synthesized EJ-AgNPs

SEM micrographs demonstrated that the green-synthesized EJ-AgNPs exhibited a predominantly spherical morphology. The elemental composition of the synthesized nanoparticles was further analyzed using Energy Dispersive X-ray Spectroscopy (EDX), as shown in Fig. 4. The nanoparticles were uniformly distributed with limited agglomeration, indicating effective stabilization during the green synthesis process. The average particle size determined from SEM micrographs was approximately 13.52 ± 1.55 nm, confirming the successful formation of nanoscale silver particles. The observed morphology and size distribution suggest that the biomolecules present in the Eriobotrya japonica seed extract played a crucial role in controlling nanoparticle growth and preventing excessive aggregation. The difference between SEM and DLS size measurements is expected, as SEM provides the physical size of dried nanoparticles, whereas DLS measures the hydrodynamic diameter of particles in suspension, including the contribution of capping agents and solvation layers.

The EDX spectrum confirmed the presence of silver (Ag) as the major constituent, along with carbon (C), oxygen (O), and chlorine (Cl), which is consistent with previously reported studies on plant-mediated silver nanoparticles [40, 41]. The atomic percentages of the detected elements are provided as an inset in Fig. 4, further supporting the successful green synthesis and stabilization of the nanoparticles. A strong characteristic signal corresponding to silver was observed at approximately 3 keV, which is a typical feature of silver nanoparticles and confirms their successful biosynthesis. Previous studies have consistently reported that AgNPs exhibit a prominent absorption peak around 3 keV in EDX spectra [42]. The detection of carbon and oxygen elements is expected in green synthesis approaches and can be attributed to the presence of organic biomolecules from the loquat seed extract used during nanoparticle synthesis.

### X-Ray Diffraction Analysis of EJ-AgNPs

The crystalline characteristics of the green-synthesized EJ-AgNPs were investigated using X-ray diffraction (XRD) analysis. As shown in Fig. 4, the diffraction pattern exhibited several distinct and well-defined peaks, confirming the formation of a crystalline nanostructure. The most intense diffraction peak was observed at 2θ ≈ 38.2°, accompanied by additional reflections at approximately 44.4°, 64.6°, and 77.5°, which can be indexed to the (111), (200), (220), and (311) crystallographic planes of face-centered cubic (fcc) metallic silver, respectively. These diffraction features are in good agreement with the standard JCPDS card No. 89–3722 and are consistent with previously reported data for green-synthesized silver nanoparticles [43]. The predominance of the (111) reflection suggests a preferred crystallographic orientation, which is commonly observed in plant-mediated silver nanoparticle systems. In addition, the noticeable broadening of the diffraction peaks indicates the formation of nanoscale crystallites. Such peak broadening may be attributed to reduced crystallite size as well as lattice strain and surface effects induced by phytochemical capping agents present in the loquat seed extract. These phytochemicals, acting as both reducing and stabilizing agents during synthesis, can influence nucleation and crystal growth processes, thereby affecting the structural properties of the nanoparticles. Furthermore, the absence of additional impurity peaks in the diffraction pattern indicates the high phase purity of the synthesized EJ-AgNPs. Overall, the XRD results confirm the crystalline nature and structural integrity of the synthesized nanoparticles and are in agreement with the morphological observations obtained from SEM analysis.

### Biological Activities

#### Antioxidant Activity of EJ-AgNPs (DPPH assay)

**Fig. 5:**
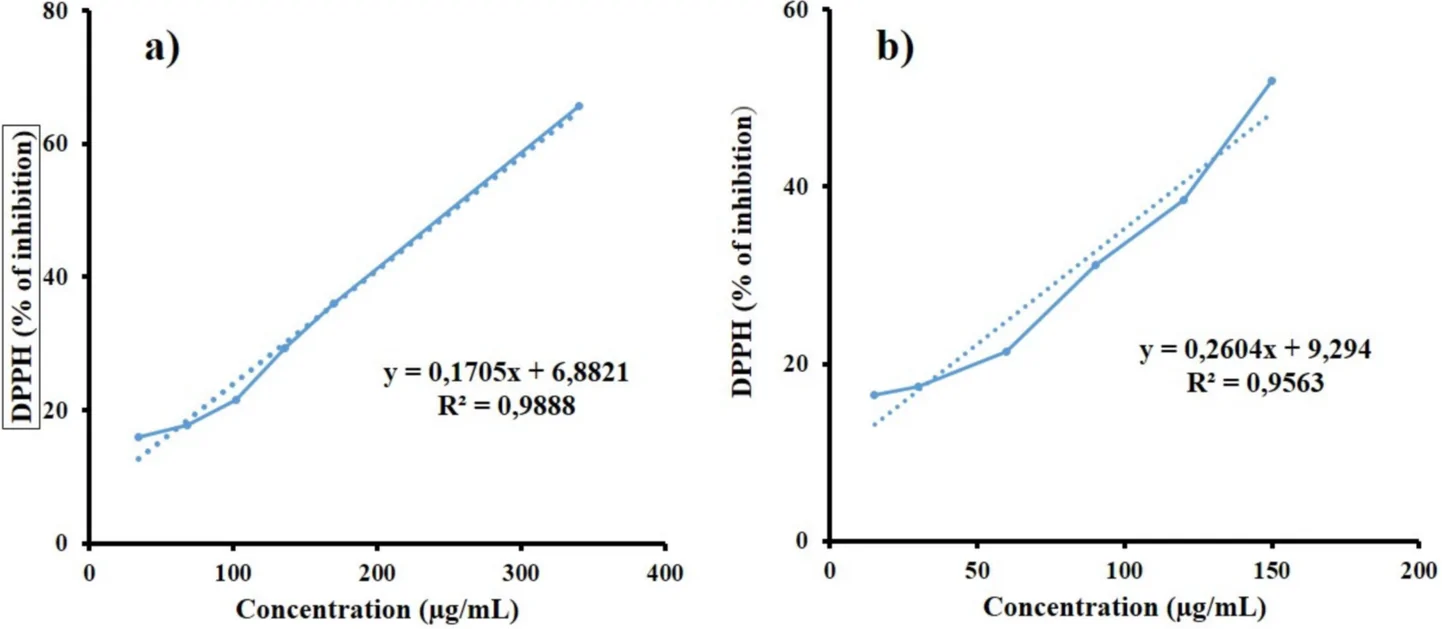
EC_50_ values of antioxidant activity for loquat seed extract (**a**) and EJ-AgNPs (**b**)

The antioxidant activity of the green-synthesized EJ-AgNPs was evaluated using the DPPH radical scavenging assay. The effective concentration required to scavenge 50% of DPPH radicals (EC_50_) was determined for both the waste loquat seed extract and the synthesized EJ-AgNPs. As shown in Fig. 5, the EC_50_ value of the loquat seed extract was calculated as 253 ± 21.8 µg/mL, whereas the EC50 value of EJ-AgNPs was significantly lower at 156.32 ± 15.8 µg/mL, indicating enhanced antioxidant activity following nanoparticle synthesis. The lower EC_50_ value observed for EJ-AgNPs demonstrates a stronger free radical scavenging capacity compared to the crude seed extract. The antioxidant activity of AgNPs is thought to be due to the presence of secondary metabolites including flavonoids with free OH groups in loquat seeds. Therefore, Ag + ion is thought to be reduced and stabilised by secondary metabolites. Similar enhancements in antioxidant performance have been reported for plant-mediated silver nanoparticles, supporting the role of green synthesis in improving bioactivity [44, 45].

Oxidative stress is widely recognized as a contributing factor in the development and progression of metabolic disorders, including diabetes mellitus, through mechanisms involving chronic inflammation and cellular damage [46]. In this regard, the pronounced antioxidant capacity observed for EJ-AgNPs provides a rational basis for further evaluation of their biological activities related to glucose metabolism. The antioxidant behavior of EJ-AgNPs may therefore be considered a supportive mechanism underlying their subsequent investigation for antidiabetic enzyme inhibitory effects, such as DPP-IV and α-glucosidase inhibition.

### DPP-IV Inhibitory Activity

**Fig. 6:**
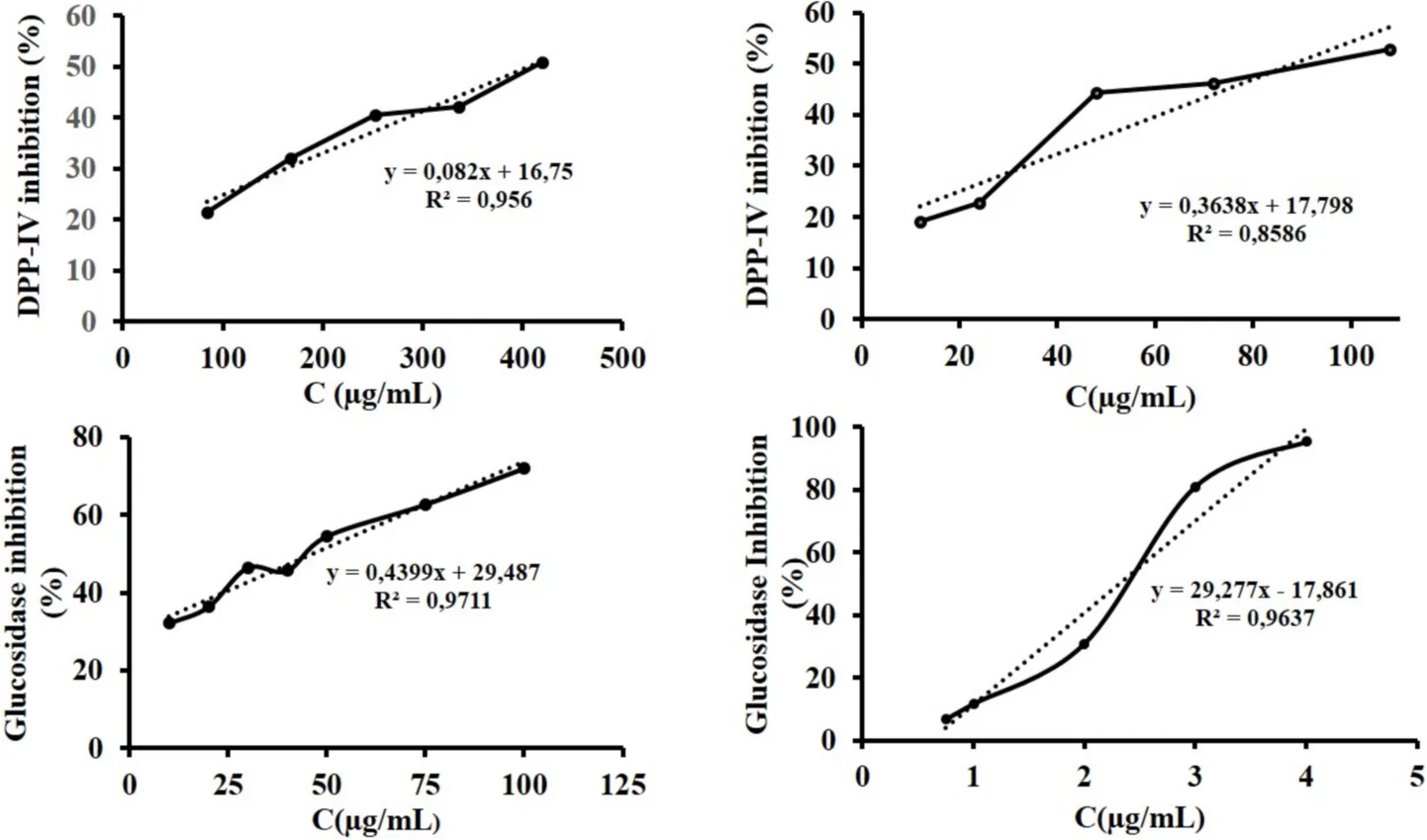
DPP-IV inhibitory activity and Glucosidase inhibitory activity of *E. japonica* waste seed extract (**a**) and EJ-AgNPs (**b**)

Dipeptidyl peptidase-IV (DPP-IV) is a key regulatory enzyme involved in glucose metabolism through the inactivation of incretin hormones, and its inhibition is considered an effective strategy for the management of type 2 diabetes mellitus [47, 48]. Therefore, the identification of natural and biocompatible DPP-IV inhibitors has gained increasing attention in recent years. In the present study, the DPP-IV inhibitory activities of Eriobotrya japonica waste seed extract and the silver nanoparticles (AgNPs) synthesized from the extract were evaluated. The IC50 values of the samples are presented in Fig. 6. The DPP-IV inhibition assay revealed that the seed extract exhibited its highest inhibitory activity at a concentration of 1.0 mg/mL, corresponding to 19.67% inhibition. In contrast, the synthesized AgNPs showed a markedly higher DPP-IV inhibitory activity, with an inhibition rate of 42.98% at the tested concentration. These findings are in good agreement with previous studies reporting significant DPP-IV inhibitory activity of plant-mediated silver nanoparticles [49, 50]. The enhanced inhibitory effect observed for AgNPs may be attributed to improved interaction between enzyme active sites and surface-bound phytochemicals, as well as the increased surface area of the nanoparticles.

### Glucosidase Inhibitory Activity

α-glucosidase is enzyme responsible for hydrolysis of disaccharides, trisaccharides, oligosaccharides, and starch. Inhibition of these enzymes decreases digestion of carbohydrates and therefore retards glucose absorption [51, 52].The α-glucosidase inhibitory activity of the Eriobotrya japonica waste seed extract and the synthesized silver nanoparticles was quantitatively evaluated based on their IC_50_ values (Fig. 6). The seed extract exhibited an IC_50_ value of 52.61 µg/mL, whereas a markedly lower IC_50_ value of 2.32 µg/mL was obtained for the green-synthesized silver nanoparticles. The substantially lower IC_50_ value of the AgNPs indicates a significantly stronger α-glucosidase inhibitory activity compared to the crude seed extract. This pronounced enhancement in inhibitory efficiency following nanoparticle synthesis may be attributed to the increased surface area of AgNPs and the improved interaction between enzyme active sites and bioactive phytochemicals immobilized on the nanoparticle surface. These findings indicate that the synthesized AgNPs exhibit strong α-glucosidase inhibitory activity and may have potential relevance in enzyme inhibition studies related to glucose metabolism. Similar results were also found in the green synthesized silver nanoparticles from other medicinal plants [53, 54].

### Antiglycation Activity

**Fig. 7:**
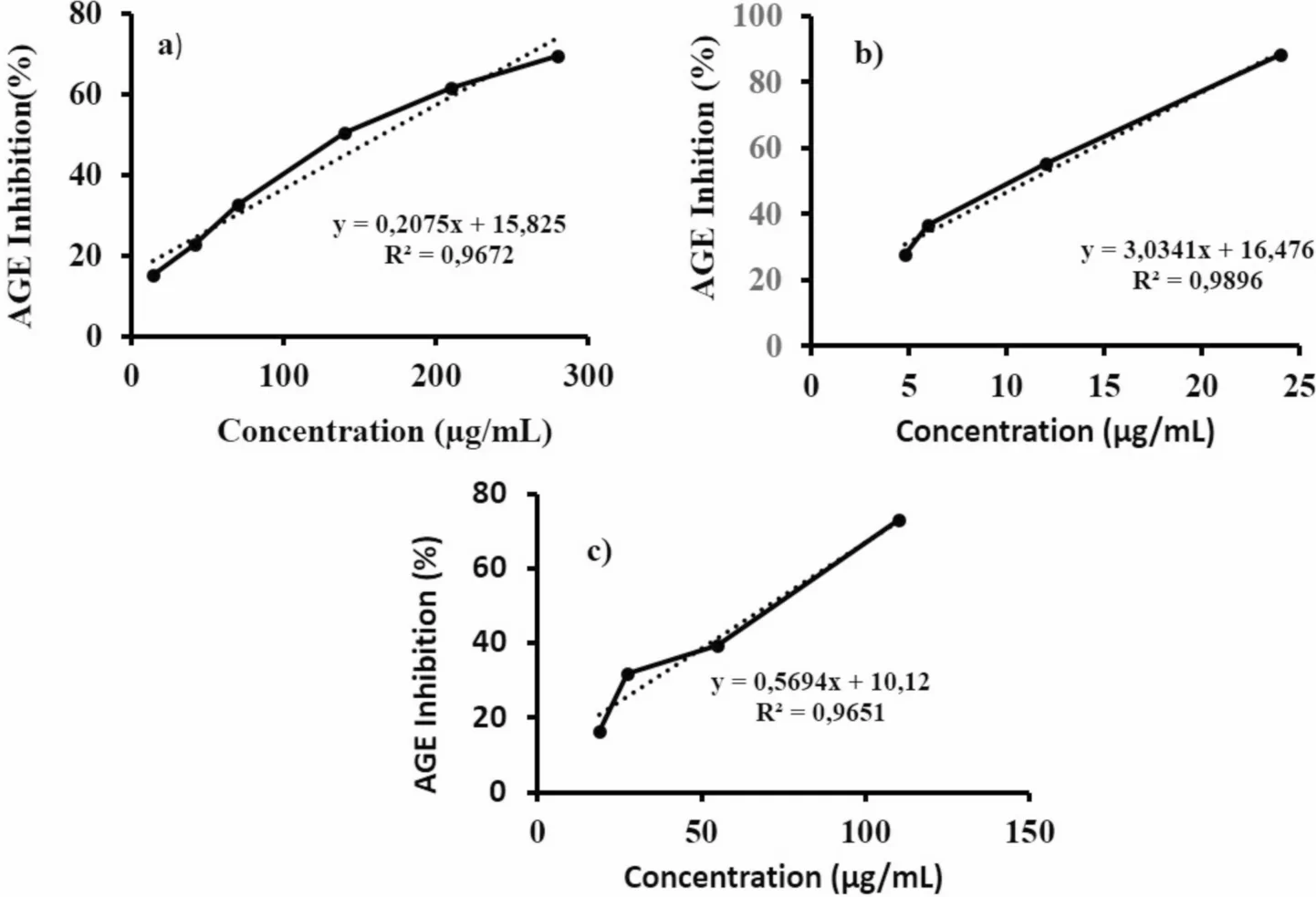
Antiglycation inhibition activity of (**a**) Eriobotrya japonica waste seed extract, (**b**) green-synthesized silver nanoparticles (AgNPs), and (**c**) aminoguanidine

The antiglycation potential of Eriobotrya japonica waste seed extract and the corresponding green-synthesized silver nanoparticles (AgNPs) was comparatively evaluated in this study. The crude seed extract exhibited a moderate antiglycation effect, with an IC₅₀ value of 164.9 µg/mL (Fig. 7). In contrast, the AgNPs synthesized using the loquat seed extract demonstrated substantially higher antiglycation activity, with a markedly lower IC_50_ value of 11.04 µg/mL. These findings suggest that nanoparticle formation enhances the antiglycation potential of the bioactive constituents present in the seed extract.

The enhanced antiglycation activity of the synthesized AgNPs may be associated with their pronounced antioxidant capacity. Compounds exhibiting strong antioxidant properties are known to suppress the formation of advanced glycation end products (AGEs) by scavenging reactive oxygen species and reactive carbonyl intermediates involved in glycation pathways [55]. In this context, the improved antioxidant activity of AgNPs may contribute to their increased antiglycation performance. The observed results are consistent with previous studies reporting enhanced antiglycation activity of plant-mediated silver nanoparticles, where nanoscale properties and phytochemical capping agents were suggested to play a key role in modulating glycation processes.

Furthermore, the biological activities observed in this study, including antioxidant, antiglycation, and enzyme inhibitory effects, may be mechanistically interconnected. Antioxidant activity contributes to the reduction of oxidative stress, which is closely associated with glycation reactions and AGE formation. In addition, phytochemical compounds associated with the nanoparticle surface may interact with enzyme active sites, contributing to α-glucosidase and DPP-IV inhibition. These combined effects suggest a possible synergistic relationship between antioxidant capacity, antiglycation potential, and enzyme inhibition, although the precise molecular mechanisms remain to be clarified.

It should also be noted that plant-mediated green synthesis approaches may be influenced by variations in phytochemical composition. Factors such as geographical origin, seasonal variation, plant maturity, and extraction conditions can affect the composition of bioactive compounds, which in turn may influence nanoparticle synthesis efficiency and biological activity. Therefore, standardization of extraction procedures and detailed characterization of phytochemical content are important for improving reproducibility and consistency [56, 57].

In addition to their promising biological activities, the potential toxicity and environmental impact of silver nanoparticles should be carefully considered. Although plant-mediated green synthesis is generally associated with improved biocompatibility due to the presence of surface-bound phytochemicals, silver nanoparticles may still exhibit dose-dependent cytotoxic effects and ecological risks. Previous studies have reported that factors such as particle size, concentration, surface chemistry, and exposure conditions can significantly influence the toxicity profile of AgNPs. Therefore, a comprehensive evaluation of biocompatibility and environmental safety is essential for their potential biomedical applications. Recent studies have emphasized the importance of detailed toxicity assessments and risk analysis for nanomaterials to ensure their safe and responsible use [58].

## Conclusion

In this study, waste Eriobotrya japonica seeds were successfully valorized as a sustainable biomass resource for the green synthesis of silver nanoparticles (AgNPs). The green synthesis approach enabled the production of stable and well-characterized nanoparticles, demonstrating the effective utilization of agricultural waste for the generation of value-added nanomaterials. The synthesized nanoparticles exhibited enhanced biological activities compared to the crude seed extract, including antioxidant, antiglycation, and enzyme inhibitory effects. In particular, the AgNPs showed stronger α-glucosidase and DPP-IV inhibitory activities, along with improved antiglycation potential, as reflected by lower IC_50_ values. These findings suggest that nanoparticle formation may enhance the biofunctional properties of plant-derived materials, potentially through the combined effects of the metallic core and surface-bound phytochemicals. However, it should be emphasized that the present findings are based solely on in vitro biochemical assays, and no direct conclusions regarding therapeutic efficacy can be drawn. In addition, the safety profile, cytotoxicity, biocompatibility, and detailed molecular mechanisms of action of the synthesized nanoparticles were not evaluated within the scope of the current study. Overall, the findings highlight the potential of waste loquat seeds as a renewable resource for green nanoparticle synthesis and contribute to the growing field of biomass valorization and sustainable nanotechnology.

## Data Availability

All the data generated or analysed during this are included in this article.

## Declarations

### Conset for Publication

All authors gave their consent for publication.
